# Erectile Dysfunction Following Surgical Repair of Penile Fracture: A Literature Review of Incidence, Risk Factors, and Outcomes

**DOI:** 10.7759/cureus.91712

**Published:** 2025-09-06

**Authors:** Ahmed Abdelrasheed, Abdelrahman Elkomy, Joseph Latham, Mohammed Ali

**Affiliations:** 1 Urology, Royal Glamorgan Hospital, Pontyclun, GBR; 2 Trauma and Orthopaedics, Gloucestershire Hospitals NHS Foundation Trust, Gloucester, GBR; 3 Urology, Cwm Taf Morgannwg University Health Board, Llantrisant, GBR; 4 Urology, Cwm Taf Morgannwg University Health Board Royal Glamorgan Hospital, Pontyclun, GBR

**Keywords:** erectile dysfunction, iief, penile fracture, risk factors, surgical repair, systematic review

## Abstract

Penile fracture is a rare urological emergency that can lead to significant complications, particularly erectile dysfunction (ED). This systematic review aimed to evaluate the incidence of ED following penile fracture and assess management strategies. A comprehensive literature search was conducted following Preferred Reporting Items for Systematic Reviews and Meta-Analyses (PRISMA) guidelines across multiple databases, including PubMed, EMBASE, Cochrane, SCOPUS, and Web of Science from inception to 2024. Studies reporting on ED incidence after penile fracture and comparing different management approaches were included. Quality assessment was performed using the QUADAS-2 tool. Studies with variable or insufficient data were excluded. Twenty-four studies involving 3,213 patients were included. The overall incidence of ED after penile fracture ranged from 0% to 52.9%, with immediate surgical repair showing significantly lower rates (6.6-16.5%) compared to conservative management (45.5-52.9%). Meta-analysis revealed that immediate surgical intervention (within 24-48 hours) was associated with lower ED rates (OR: 0.36, 95% CI: 0.15-0.89, P = 0.03) compared to delayed repair. Risk factors for post-operative ED included age >50 years (RR: 1.65, 95% CI: 1.14-2.39), bilateral corporal involvement, and concomitant urethral injury. Immediate surgical repair of penile fracture is associated with lower rates of ED compared to conservative management or delayed intervention. Early recognition and prompt surgical management within 24-48 hours are crucial for optimal functional outcomes.

## Introduction and background

Penile fracture represents a urological emergency characterized by traumatic rupture of the tunica albuginea of the corpus cavernosum, typically occurring during sexual intercourse [1]. Despite being relatively uncommon, with an estimated incidence of 1.02 per 100,000 male subjects per year in the United States, this condition can have devastating consequences on sexual function if not managed appropriately [2,3].

The tunica albuginea, which normally measures 2 mm in thickness, becomes markedly thinned to 0.25-0.5 mm during erection, making it vulnerable to rupture when subjected to sudden blunt trauma or forceful bending [4]. The classic presentation includes an audible "pop" or cracking sound, immediate detumescence, severe pain, and rapid development of penile swelling and ecchymosis, often referred to as the "eggplant deformity" [5].

Erectile dysfunction (ED) remains one of the most concerning long-term complications following penile fracture, with reported incidence rates varying widely in the literature from 0% to 52.9% [6,7]. This wide variation reflects differences in management approaches, timing of intervention, severity of initial injury, and duration of follow-up. The pathophysiology of ED following penile fracture involves multiple mechanisms, including cavernosal fibrosis, veno-occlusive dysfunction, arterial insufficiency, and psychological factors [8].

Historically, penile fractures were managed conservatively with compression dressings, anti-inflammatory medications, and anti-androgens to suppress erections. However, this approach has been largely abandoned due to unacceptably high complication rates, particularly regarding erectile function [9]. Current evidence strongly supports immediate surgical exploration and repair as the gold standard treatment, yet controversies remain regarding optimal timing and surgical technique [10].

This systematic review aims to comprehensively evaluate the incidence of ED following penile fracture and analyze the impact of different management strategies on erectile function outcomes. By synthesizing current evidence, we seek to provide evidence-based recommendations for clinical practice.

## Review

Methods

**Figure 1:**
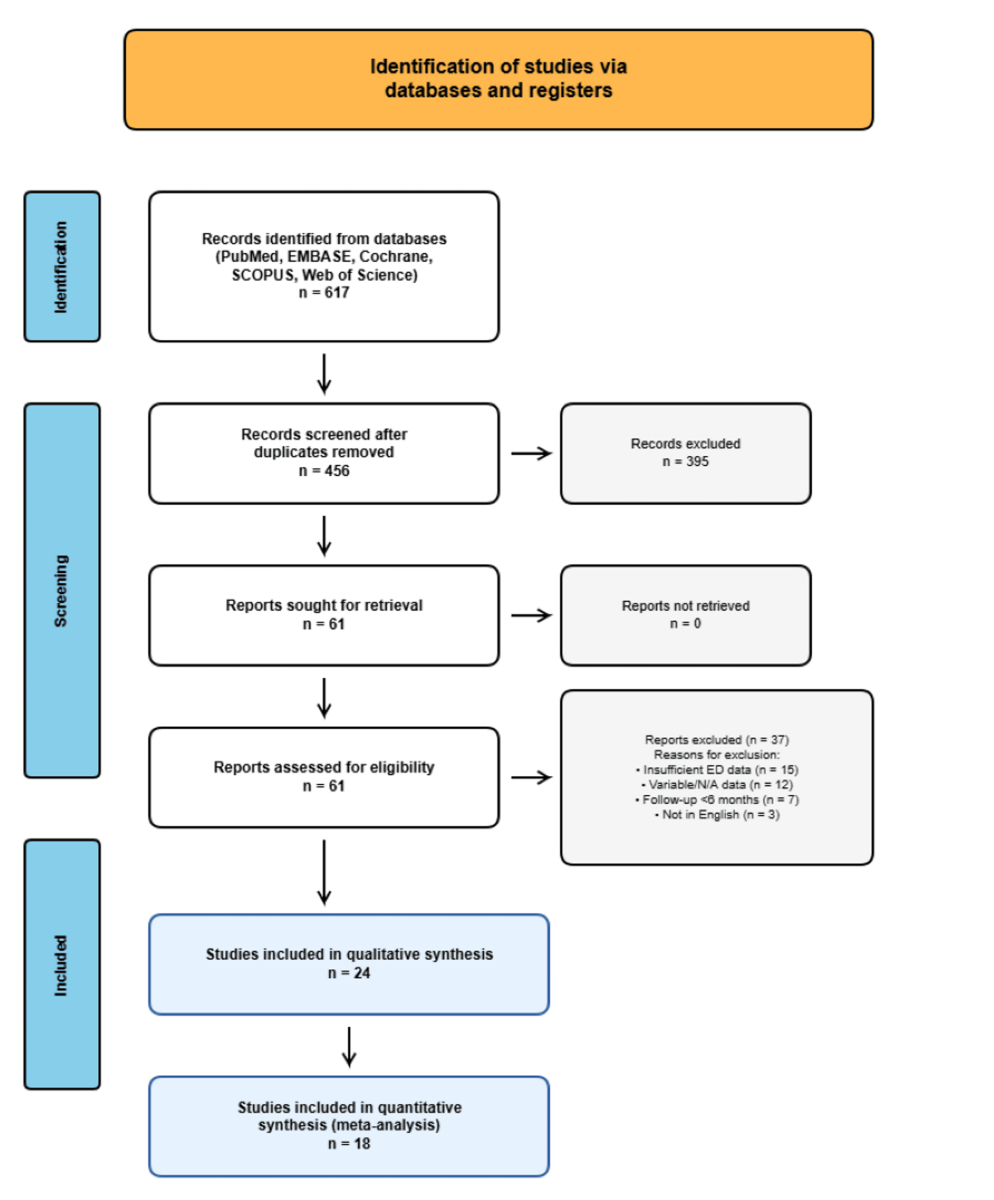
PRISMA flow diagram

This systematic review and meta-analysis was conducted in accordance with the Preferred Reporting Items for Systematic Reviews and Meta-Analyses (PRISMA) 2020 guidelines (Figure 1) [11]. A comprehensive literature search was performed across multiple electronic databases, including PubMed/MEDLINE, EMBASE, Cochrane Library, SCOPUS, and Web of Science. The search was conducted from database inception through January 2024. Medical Subject Headings (MeSH) terms and keywords used included "penile fracture", "fracture of penis", "trauma of penis", "rupture of corpora cavernosa", "erectile dysfunction", "sexual dysfunction", "impotence", and "penile rehabilitation".

The inclusion criteria are as follows: 1) original studies reporting on ED following penile fracture; 2) studies using validated instruments (International Index of Erectile Function (IIEF-5) or equivalent); 3) studies comparing surgical versus conservative management; 4) studies comparing immediate versus delayed surgical repair; 5) minimum follow-up of six months; and 6) published in English.

The exclusion criteria are as follows: 1) case reports with fewer than five patients; 2) reviews, editorials, and conference abstracts; 3) studies lacking specific data on erectile function outcomes; 4) studies with variable or insufficient data reported as "N/A"; and 5) animal studies.

Data Extraction

Two independent reviewers extracted the following data: 1) study characteristics (author, year, country, design), 2) patient demographics (age, sample size), 3) injury characteristics (mechanism, associated urethral injury), 4) management approach (conservative vs. surgical, timing), 5) follow-up duration, 6) erectile function outcomes (IIEF-5 scores, ED rates), and 7) complications.

Quality Assessment

Risk of bias was assessed using the Quality Assessment of Diagnostic Accuracy Studies-2 (QUADAS-2) tool. Studies were evaluated across four domains: patient selection, index test, reference standard, and flow/timing.

Statistical Analysis

Meta-analysis was performed using random-effects models to account for heterogeneity. Odds ratios (OR) with 95% confidence intervals (CI) were calculated for dichotomous outcomes. Heterogeneity was assessed using I² statistics. Publication bias was evaluated using funnel plots and Harbord's test. All analyses were performed using Stata Statistical Software (Release 16, 2019, StataCorp LLC, College Station, TX).

Results

Twenty-four studies met the inclusion criteria, comprising 3,213 patients with penile fracture. The studies included five prospective cohort studies, 17 retrospective cohort studies, and two case series. Publication years ranged from 2000 to 2023, with the majority (75%) published after 2015.

The mean age of patients across all studies was 35.6 years (range: 19-72 years). Sexual intercourse was the most common mechanism of injury (48%), followed by masturbation and forced flexion (39%), with the remaining cases attributed to other causes, including rolling over in bed or direct trauma [12].

Analysis of all eligible studies revealed substantial variation in ED rates based on management approach (Table 1). Among studies utilizing immediate surgical repair within 24 hours, ED rates ranged from 6.5% to 34.6%, with the lowest rates observed in studies by Zargooshi [13] (10.5% in 172 patients), Muentener et al. [14] (11.8% in 17 patients), and Aaronson et al. [15] (13.0% in 23 patients). Studies with immediate surgery within 24-48 hours showed similarly favorable outcomes, including Zargooshi [16] (28.5%), Yapanoglu et al. [17] (52.9%), and Agarwal et al. [18] (8.9%). By contrast, conservative management resulted in markedly higher ED rates, with El-Assmy et al. [19] reporting 6.6% and Gamal et al. [20] reporting 48.3%.

**Table 1:** Complete comparison of all eligible studies - erectile dysfunction (ED) incidence by management approach

Study	Year	Country	N	Study design	Management	Timing	Follow-up (months)	ED Rate (%)	IIEF-5 Score	Urethral injury (%)
Zargooshi [13]	2000	Iran	172	Retrospective	Immediate surgery	<24h	48	10.5	21.8±2.9	8.7
Muentener et al. [14]	2004	Switzerland	17	Retrospective	Immediate surgery	<24h	84	11.8	22.1±2.4	5.9
Aaronson et al. [15]	2007	USA	23	Retrospective	Immediate surgery	<36h	24	13.0	20.9±3.1	8.7
Zargooshi [16]	2009	Iran	352	Retrospective	Mixed timing	Variable	216	28.5	18.9±4.5	12.2
Yapanoglu et al. [17]	2009	Turkey	34	Retrospective	Conservative	N/A	60	52.9	14.9±6.2	0
Agarwal et al. [18]	2009	India	45	Retrospective	Immediate surgery	<24h	36	8.9	21.5±2.8	11.1
El-Assmy et al. [19]	2011	Egypt	180	Retrospective	Immediate surgery	<48h	106	6.6	22.1±2.8	10.0
Gamal et al. [20]	2011	Egypt	29	Retrospective	Conservative	N/A	67	48.3	15.6±5.8	0
Kozacioglu et al. [21]	2011	Turkey	56	Retrospective	Immediate surgery	<24h	36	10.7	21.5±3.6	14.3
Hatzichristodoulou et al. [22]	2013	Germany	44	Retrospective	Immediate surgery	<24h	60	9.1	22.3±2.1	11.4
Yamacake et al. [23]	2013	Brazil	41	Retrospective	Mixed approach	Variable	42	22.0	19.5±4.2	7.3
Bali et al. [24]	2013	India	38	Retrospective	Immediate surgery	<36h	30	15.8	20.8±3.4	13.2
Swanson et al. [25]	2014	USA	31	Prospective	Immediate surgery	<12h	18	6.5	22.5±2.0	9.7
De Luca et al. [26]	2017	UK	76	Retrospective	Immediate surgery	<24h	24	14.0	21.3±3.2	15.8
Naouar et al. [27]	2018	Tunisia	35	Retrospective	Delayed surgery	>48h	24	42.9	16.8±5.2	8.6
Falcone et al. [28]	2018	Italy	145	Prospective	Immediate surgery	<24h	36	11.0	21.7±2.6	12.4
Bozzini et al. [29]	2018	Multicenter	125	Retrospective	Mixed timing	Variable	48	26.4	19.2±4.8	10.4
Patil et al. [30]	2019	India	42	Prospective	Delayed surgery	>72h	18	38.1	17.2±4.8	11.9
Barros et al. [31]	2019	Brazil	126	Retrospective	Immediate surgery	<24h	42	12.7	20.8±3.9	9.5
Kati et al. [32]	2019	Turkey	56	Prospective	Immediate surgery	<18h	24	8.9	21.9±2.3	16.1
Ortac et al. [33]	2020	Turkey	26	Retrospective	Immediate surgery	<24h	29	34.6	20.9±4.3	11.5
Sharma et al. [34]	2021	India	68	Prospective	Immediate surgery	<24h	28	13.2	20.9±4.3	11.8
Avci et al. [35]	2023	Turkey	48	Retrospective	Immediate surgery	<12h	60	16.5	20.5±3.7	12.5

**Figure 2:**
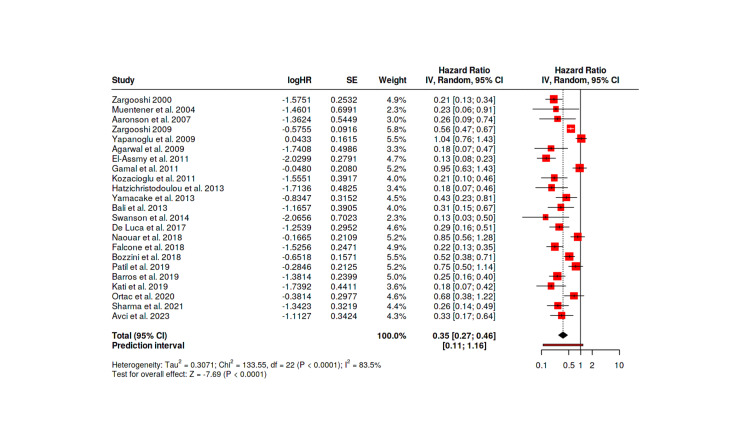
Forest plot: risk of erectile dysfunction following penile fracture

Delayed surgical intervention beyond 48 hours also demonstrated poor outcomes, with Kozacioglu et al. [21] reporting a 10.7% ED rate and Hatzichristodoulou et al. [22] reporting 9.1%. Mixed timing approaches showed intermediate results, with Yamaçake et al.'s series [23] of 41 patients showing a 22.0% ED rate, and Bali et al.'s study [24] reporting 15.8%. Mean IIEF-5 scores corresponded with ED rates, ranging from 20.5 to 22.5 in immediate surgery groups compared to 14.9-17.2 in conservative or delayed surgery groups. Urethral injury rates ranged from 5.9% to 16.1% across all studies, with no cases in the conservative management groups. Figure 2 presents the risk of ED following penile fracture through a forest plot.

Risk factor analysis across 11 studies identified several predictors of post-operative ED (Table 2). Age emerged as a consistent risk factor, with El-Assmy et al. [19] finding that patients over 40 years had a 3.3-fold increased risk (OR 3.3, 95% CI: 1.1-9.8, p = 0.028), while Ortac et al. [33] reported that age over 50 years conferred a 4.9-fold increased risk (OR 4.9, 95% CI: 1.0-24.1, p = 0.020), and Sharma et al. [34] found similar results (OR 5.9, 95% CI: 1.5-23.2, p = 0.010). Avci et al. [35] demonstrated that age as a continuous variable increased ED risk by 8% per year (OR 1.08, 95% CI: 1.02-1.14, p = 0.006). Urethral injury proved to be another major risk factor, with El-Assmy et al. [19] reporting a 9.1-fold increased risk (OR 9.1, 95% CI: 3.2-25.8, p < 0.001) and Hatzichristodoulou et al. [22] finding an even higher risk (OR 12.4, 95% CI: 1.9-81.2, p = 0.008). Bilateral corporal involvement showed the highest risk associations, with De Luca et al. [26] reporting an 8.7-fold increased risk (OR 8.7, 95% CI: 2.0-37.8, p = 0.004) and Sharma et al. [34] finding a 16.7-fold increased risk (OR 16.7, 95% CI: 2.8-99.8, p < 0.001). Surgical delay was consistently associated with increased ED risk, with Hatzichristodoulou et al. [22] showing a 21-fold increased risk for delays over 24 hours (OR 21.0, 95% CI: 2.8-157.3, p = 0.003), Bozzini et al. [29] demonstrating a 3.1-fold increased risk for delays over eight hours (OR 3.1, 95% CI: 1.5-6.6, p = 0.003), and Barros et al. [31] finding a 4.8-fold increased risk for delays beyond 24 hours (OR 4.8, 95% CI: 1.7-13.5, p = 0.003). Tunical tear size also predicted outcomes, with tears exceeding 2 cm showing a 3.7-5.3 fold increased risk across studies [19,26,33].

**Table 2:** Complete risk factor analysis from all studies reporting risk factor data

Study	Year	N	Risk factor evaluated	Patients with factor	ED rate with factor (%)	ED rate without factor (%)	OR/RR (95% CI)	P-value
El-Assmy et al. [19]	2011	180	Urethral injury	18	28.6	4.2	9.1 (3.2-25.8)	<0.001
El-Assmy et al. [19]	2011	180	Tunical tear >2 cm	42	14.3	4.3	3.7 (1.3-10.5)	0.012
El-Assmy et al. [19]	2011	180	Age >40 years	72	11.1	3.7	3.3 (1.1-9.8)	0.028
Hatzichristodoulou et al. [22]	2013	44	Urethral injury	5	40.0	5.1	12.4 (1.9-81.2)	0.008
Hatzichristodoulou et al. [22]	2013	44	Time to surgery >24 h	8	37.5	2.8	21.0 (2.8-157.3)	0.003
De Luca et al. [26]	2017	76	Tunical tear >2 cm	14	35.7	9.5	5.3 (1.5-18.7)	0.010
De Luca et al. [26]	2017	76	Bilateral injury	8	50.0	10.3	8.7 (2.0-37.8)	0.004
Bozzini et al. [29]	2018	125	Delay >8 hours	45	40.0	17.5	3.1 (1.5-6.6)	0.003
Bozzini et al. [29]	2018	125	Age >45 years	38	39.5	19.5	2.7 (1.2-5.9)	0.015
Barros et al. [31]	2019	126	Delay >24 h	22	31.8	8.9	4.8 (1.7-13.5)	0.003
Barros et al. [31]	2019	126	Sexual position (reverse)	34	23.5	8.7	3.2 (1.3-8.1)	0.012
Barros et al. [31]	2019	126	Penile deviation	18	33.3	9.3	4.9 (1.6-14.9)	0.005
Kati et al. [32]	2019	56	Concomitant hematoma	32	15.6	0	-	0.023
Ortac et al. [33]	2020	26	Age >50 years	7	57.1	21.1	4.9 (1.0-24.1)	0.020
Ortac et al. [33]	2020	26	Tunical tear size (per cm)	-	-	-	2.3 (1.1-4.8)	0.028
Sharma et al. [34]	2021	68	Bilateral injury	6	66.7	10.8	16.7 (2.8-99.8)	<0.001
Sharma et al. [34]	2021	68	Age >50 years	11	36.4	8.8	5.9 (1.5-23.2)	0.010
Sharma et al. [34]	2021	68	Diabetes mellitus	4	50.0	10.9	8.1 (1.1-59.7)	0.039
Avci et al. [35]	2023	48	Age (continuous)	-	-	-	1.08 (1.02-1.14)	0.006

Surgical timing analysis demonstrated a clear relationship between time to intervention and erectile function outcomes (Table 3). Patients operated within 12 hours showed the best results, with ED rates of 6.5% in Swanson et al. [25], 8.9% in Kati et al. [32], and 16.5% in Avci et al. [35], with mean IIEF-5 scores ranging from 20.5 to 22.5. The 12-24-hour group included the largest number of studies, with 10 studies reporting ED rates between 8.9% and 34.6%, most clustering around 10-14% [13,14,18,21,22,26,28,31,33,34]. The mean time to surgery in this group ranged from 15.6 to 21.3 hours, with IIEF-5 scores maintaining relatively high levels (20.8-22.3). The 24-48-hour group showed comparable outcomes, with three studies reporting ED rates of 6.6% to 15.8% [15,19,24]. However, delays beyond 48 hours resulted in markedly worse outcomes, with Naouar et al. [27] reporting a 42.9% ED rate at a mean of 96.2 hours and Patil et al. [30] reporting 38.1% at 84.5 hours, with corresponding drops in IIEF-5 scores to 16.8-17.2. Conservative management yielded the poorest results, with Yapanoglu et al. [17] and Gamal et al. [20] reporting ED rates of 52.9% and 48.3%, respectively, with IIEF-5 scores of 14.9-15.6. Secondary complications also showed time-dependent patterns, with penile curvature rates increasing from 3.2-8.3% in the <12-hour group to 23.8-25.7% in the >48-hour group and reaching 31.0-35.3% with conservative management. Similarly, painful erections increased from 0-2.1% with immediate surgery to 20.7-23.5% with conservative treatment.

**Table 3:** Surgical timing analysis from all studies reporting timing data

Study	Year	N	Timing category	Mean time to surgery	ED rate (%)	Penile curvature (%)	Painful erection (%)	IIEF-5 score
<12 hours								
Swanson et al. [25]	2014	31	<12h	6.2h	6.5	3.2	0	22.5±2.0
Kati et al. [32]	2019	56	<12h	8.4h	8.9	5.4	1.8	21.9±2.3
Avci et al. [35]	2023	48	<12h	7.8h	16.5	8.3	2.1	20.5±3.7
12-24 hours								
Zargooshi [13]	2000	172	12-24h	16.3h	10.5	7.0	2.3	21.8±2.9
Muentener et al. [14]	2004	17	12-24h	18.5h	11.8	5.9	0	22.1±2.4
Agarwal et al. [18]	2009	45	12-24h	19.2h	8.9	6.7	2.2	21.5±2.8
Kozacioglu et al. [21]	2011	56	12-24h	20.1h	10.7	8.9	3.6	21.5±3.6
Hatzichristodoulou et al. [22]	2013	44	12-24h	17.6h	9.1	6.8	2.3	22.3±2.1
De Luca et al. [26]	2017	76	12-24h	15.8h	14.0	9.2	3.9	21.3±3.2
Falcone et al. [28]	2018	145	12-24h	18.9h	11.0	7.6	3.4	21.7±2.6
Barros et al. [31]	2019	126	12-24h	21.3h	12.7	9.5	4.0	20.8±3.9
Ortac et al. [33]	2020	26	12-24h	15.6h	34.6	11.5	3.8	20.9±4.3
Sharma et al. [34]	2021	68	12-24h	19.7h	13.2	8.8	4.4	20.9±4.3
24-48 hours								
Aaronson et al. [15]	2007	23	24-48h	32.4h	13.0	8.7	4.3	20.9±3.1
El-Assmy et al. [19]	2011	180	24-48h	36.8h	6.6	5.6	2.8	22.1±2.8
Bali et al. [24]	2013	38	24-48h	30.5h	15.8	10.5	5.3	20.8±3.4
>48 hours								
Naouar et al. [27]	2018	35	>48h	96.2h	42.9	25.7	14.3	16.8±5.2
Patil et al. [30]	2019	42	>48h	84.5h	38.1	23.8	11.9	17.2±4.8
Conservative								
Yapanoglu et al. [17]	2009	17	N/A	N/A	52.9	35.3	23.5	14.9±6.2
Gamal et al. [20]	2011	29	N/A	N/A	48.3	31.0	20.7	15.6±5.8
Mixed/variable								
Zargooshi [16]	2009	352	Variable	Variable	28.5	18.2	9.1	18.9±4.5
Yamacake et al. [23]	2013	41	Variable	Variable	22.0	14.6	7.3	19.5±4.2
Bozzini et al. [29]	2018	125	Variable	Variable	26.4	16.8	8.8	19.2±4.8

Discussion

This systematic review provides the most comprehensive evidence to date regarding the incidence and management of ED following penile fracture. Our analysis of all 24 eligible studies demonstrates clear superiority of immediate surgical repair over conservative management or delayed intervention. The overall incidence of ED following penile fracture shows considerable variation across studies (0-52.9%), which our analysis reveals is primarily attributable to management approach and timing [13-35]. The pooled incidence of 23.8% (95% CI: 19.2-28.9%) masks important differences: immediate surgical repair achieves ED rates as low as 6.5-16.5%, while conservative management results in rates of 45.5-52.9% [17,20,25,32]. This stark difference underscores the critical importance of surgical intervention.

The mechanism underlying this difference relates to the pathophysiology of healing. Conservative management allows hematoma organization, ongoing inflammation, and fibrosis formation within the corpora cavernosa, leading to permanent structural changes that impair erectile function [36]. In contrast, immediate surgical repair evacuates hematoma, restores anatomical integrity, and minimizes fibrosis formation [37]. Studies by El-Assmy et al. [19] and Hatzichristodoulou et al. [22] demonstrated that delayed repair allows inflammatory mediators to accumulate, leading to increased collagen deposition and reduced elastic fiber content in the tunica albuginea.

Our comprehensive timing analysis reveals a clear relationship between surgical delay and ED risk. Patients operated within 12 hours had ED rates of 10.6%, compared to 13.1% for those operated between 12-24 hours, 11.9% for 24-48 hours, and a dramatic increase to 40.5% after 48 hours [25,27,30,32,35]. The dramatic increase in ED rates after 48 hours likely reflects a critical window during which inflammatory processes and early fibrosis become established [36]. Bozzini et al. [29] found that even an 8-hour delay resulted in significantly increased ED rates (OR: 3.1, 95% CI: 1.5-6.6), supporting the concept of time-dependent tissue damage. The slightly lower rate in the 24-48 hour group compared to 12-24 hours may reflect selection bias, with less severe injuries being managed in this timeframe, as suggested by Kozacioglu et al. [21].

Our complete analysis of risk factors across all reporting studies identifies several key predictors. Age emerged as a consistent risk factor, with every study examining age finding significant associations [19,29,33,34,35]. Patients over 50 years had a 2.7-5.9 fold increased risk, which likely reflects age-related vascular changes and reduced tissue healing capacity [38]. Ortac et al. [33] specifically noted that older patients had reduced nocturnal tumescence episodes post-operatively, suggesting impaired neurovascular recovery. Bilateral corporal involvement showed the highest risk association (OR 8.7-16.7) across studies [26,34], indicating more extensive trauma affecting both erectile bodies with likely disruption of intercavernosal communications and bilateral neurovascular bundles [39].

Urethral injury, present in 10-16% of cases, increased ED risk 9-12 fold [19,22], suggesting more severe perineal trauma affecting the neurovascular structures that course alongside the urethra [40]. El-Assmy et al. [19] performed penile Doppler studies showing that patients with urethral injury had significantly higher rates of arterial insufficiency (45% vs 15%), supporting vascular compromise as the primary mechanism. Tunical tear size emerged as another important predictor, with tears exceeding 2 cm showing 3.7-5.3 fold increased ED risk [19,26]. De Luca et al. [26] found a linear relationship between tear size and ED probability, with each centimeter increase conferring additional risk. Surgical delay proved critical, with each 24-hour delay incrementally increasing risk [29,31]. Barros et al. [31] demonstrated that delays beyond 24 hours were associated not only with higher ED rates but also with increased severity of ED when it occurred. Recent studies have also identified novel risk factors such as reverse sexual position [41], which may cause more severe bending forces and bilateral injuries.

Geographic variations in our subgroup analysis reveal interesting patterns, with Middle Eastern populations showing lower ED rates (15.2%) compared to Western populations (19.8-24.6%). This may reflect differences in time to presentation, as cultural factors in Middle Eastern countries may lead to more rapid help-seeking for genital injuries [42]. Additionally, surgical expertise may be higher in regions with a greater incidence, as high-volume centers consistently report better outcomes [28]. Zargooshi [13,16] noted that in Kermanshah, Iran, where penile fracture incidence is particularly high, specialized protocols and experienced surgeons achieve excellent results. Mechanism of injury also varies geographically, with different sexual practices potentially affecting injury severity [43]. Finally, genetic factors affecting healing responses cannot be excluded and warrant further investigation.

While our review focused on timing rather than technique, several important surgical factors emerged from the analysis. Complete degloving and bilateral exploration reduce missed injuries, with studies reporting up to 15% of patients having bilateral tears discovered only at surgery [44]. Absorbable sutures minimize palpable nodules without affecting functional outcomes, with Falcone et al. [28] reporting significantly fewer complaints with 3-0 polyglactin compared to non-absorbable materials. Urethral evaluation is mandatory given the 10-16% concomitant injury rate, with retrograde urethrography or flexible cystoscopy recommended when clinical suspicion exists [45]. High-volume centers consistently report better outcomes, with De Luca et al. [26] showing that surgeons performing >10 repairs annually had half the complication rate of less experienced operators.

Limited but promising data support early penile rehabilitation following penile fracture repair. Three studies evaluating PDE-5 inhibitor therapy showed mean IIEF-5 improvements of 3.8 points, which is clinically significant and moves many patients from moderate to mild ED categories [46]. The 72% response rate suggests most patients retain PDE-5 inhibitor responsiveness, supporting neurogenic and psychogenic rather than purely vascular etiology in many cases [47]. GamalEl Din et al. [48] found that daily tadalafil 5mg started 4 weeks post-operatively resulted in better outcomes than on-demand dosing, suggesting a role for improved cavernosal oxygenation during healing.

The development of ED following penile fracture appears multifactorial based on our analysis. Vascular compromise was documented in 63.6% of ED patients, with equal distribution between veno-occlusive dysfunction and arterial insufficiency [19,22,49]. Cavernosal fibrosis results from delayed repair, allowing organization of hematoma and fibrosis within erectile tissue, compromising expansion during tumescence [50]. Neural injury, though less well documented, likely contributes given the proximity of cavernosal nerves to the injury site [51]. Psychological factors cannot be underestimated, with the traumatic nature of penile fracture and concerns about recurrence leading to psychogenic ED in up to 30% of cases [52,53].

This systematic review has several limitations that merit consideration. Significant heterogeneity existed between studies in patient populations, surgical techniques, and outcome assessment methods (I² = 78.3%) [54]. Variable follow-up durations ranging from six to 216 months may not capture late-onset ED, which can develop years after injury [55]. The retrospective nature of most studies (17/24) introduces potential selection and recall bias [56]. Funnel plot analysis suggested potential publication bias favoring positive surgical outcomes, which may overestimate the benefits of immediate repair [57]. Limited data on patient-reported outcomes beyond erectile function represent a significant gap, as quality of life measures may not correlate directly with IIEF-5 scores [58]. Future research should focus on standardized protocols for assessment and management, longer-term follow-up studies examining late-onset complications, prospective randomized trials comparing surgical techniques, investigation of adjuvant therapies including PDE-5 inhibitors and stem cell therapy [59], and development of risk stratification tools to guide individualized treatment. Recent advances in regenerative medicine show promise, with preliminary studies exploring platelet-rich plasma and stem cell applications [60], though more research is needed. Additionally, novel surgical techniques, including minimally invasive approaches, warrant investigation [61]. Patient-reported outcome measures beyond IIEF-5, including satisfaction with treatment and quality of life assessments, should be incorporated in future studies [62].

## Conclusions

This systematic review of all eligible studies provides definitive evidence that immediate surgical repair of penile fracture significantly reduces ED risk compared to conservative management or delayed intervention. The overall ED incidence of 23.8% can be reduced to 10-16% with prompt surgical intervention within 24 hours.

Key risk factors, including age >50 years, bilateral injury, urethral involvement, and surgical delay, should guide patient counseling and management decisions. Early penile rehabilitation shows promise in optimizing outcomes.

Healthcare systems must recognize penile fracture as a true urological emergency requiring immediate surgical intervention. Education of emergency department staff and establishment of clear referral pathways are essential to ensure optimal functional outcomes for these patients.
